# The Use of a Pressure-Indicating Sensor Film to Provide Feedback upon Hydrogel-Forming Microneedle Array Self-Application ***In Vivo***

**DOI:** 10.1007/s11095-016-2032-z

**Published:** 2016-09-15

**Authors:** Eva M. Vicente-Pérez, Helen L. Quinn, Emma McAlister, Shannon O’Neill, Lezley-Anne Hanna, Johanne G. Barry, Ryan F. Donnelly

**Affiliations:** School of Pharmacy, Queen’s University Belfast, Medical Biology Centre, 97 Lisburn Road, Belfast, BT9 7BL UK

**Keywords:** application, feedback, microneedles, pressure-indicating sensor film, transdermal

## Abstract

**Purpose:**

To evaluate the combination of a pressure-indicating sensor film with hydrogel-forming microneedle arrays, as a method of feedback to confirm MN insertion *in vivo*.

**Methods:**

Pilot *in vitro* insertion studies were conducted using a Texture Analyser to insert MN arrays, coupled with a pressure-indicating sensor film, at varying forces into excised neonatal porcine skin. *In vivo* studies involved twenty human volunteers, who self-applied two hydrogel-forming MN arrays, one with a pressure-indicating sensor film incorporated and one without. Optical coherence tomography was employed to measure the resulting penetration depth and colorimetric analysis to investigate the associated colour change of the pressure-indicating sensor film.

**Results:**

Microneedle insertion was achieved *in vitro* at three different forces, demonstrating the colour change of the pressure-indicating sensor film upon application of increasing pressure. When self-applied *in vivo*, there was no significant difference in the microneedle penetration depth resulting from each type of array, with a mean depth of 237 μm recorded. When the pressure-indicating sensor film was present, a colour change occurred upon each application, providing evidence of insertion.

**Conclusions:**

For the first time, this study shows how the incorporation of a simple, low-cost pressure-indicating sensor film can indicate microneedle insertion *in vitro* and *in vivo*, providing visual feedback to assure the user of correct application. Such a strategy may enhance usability of a microneedle device and, hence, assist in the future translation of the technology to widespread clinical use.

## INTRODUCTION

Microneedles (MNs) are one of the most promising innovations in drug delivery, with great potential to yield tangible benefits for both patients and industry in the coming years. Since the first report of MN technology in the literature (1), a variety of manufacturing and drug delivery strategies have been described (2). The focus of the current study is on hydrogel-forming MNs, which swell on response to skin interstitial fluid, stimulating drug permeation from an attached reservoir. As the arrays are prepared from well-characterised biocompatible polymers, with the device sterility and bioburden considered, there is increasing evidence of what appears to be a convincing safety record for the hydrogel platform (3). This integrated system has been shown to deliver a range of molecules of varying molecular weight, from small molecules, such as metronidazole and ibuprofen, to larger molecules, such as insulin and bovine serum albumin (4,5). Having therefore demonstrated their promise in transdermal drug delivery, the next stage is to translate this success into patient outcomes, which will require consideration of MN use from the end-users’ perspective.

A notable benefit of MNs, compared to a conventional needle and syringe delivery system, is the opportunity for self-application, with no requirement for healthcare professional intervention (6). To ensure MN arrays can be easily self-applied by all types of patients, the usability and acceptability of any final product must be carefully considered. Knowledge of these intrinsically linked concepts can only be gained through engagement with potential end-users, ideally at the developmental stage. We have previously investigated the self-application of hydrogel-forming MN arrays, with the use of a patient information leaflet and pharmacist counselling for participant instruction (7). Although unanimously successful in MN insertion, following MN self-application, 80% of the participants reported that they were unsure if they had applied enough pressure to successfully insert the MNs. Interestingly, the same point has been raised in other studies, designed to explore theoretical views and opinions on MN technology (8–11). In these cases, the willingness of the public to self-apply MN arrays was highlighted but, simultaneously, an uncertainty regarding how they would assess their performance, noting this as important for personal assurance of drug delivery. It was, therefore, hypothesised that potential end-users would prefer the inclusion of a feedback mechanism in a MN delivery system, in order to provide confirmation that the MN array has been used appropriately. As an example, a recent study reported the use of a snap-based device for MN application, designed to provide audible feedback to the user upon MN insertion, for the purposes of influenza self-vaccination (6). The use of this response mechanism increased skin staining, used as a measure of MN insertion, from 90 to 96% on the first attempt, when compared to MN application with no feedback device. In this case, however, the method of feedback, i.e. the snapping noise, was built into the applicator device and not the array itself. Considering application of a MN array by hand, with no device to assist, the method of feedback will need to be incorporated into the patch design, in order to create a product with similar features to a conventional transdermal patch. As the contentious issue is the force to be applied, it follows that the ideal response directly relates to this parameter. The correlation between force and hydrogel-forming MN insertion has already been established (12) and, as pressure is defined as force per unit area, the pressure applied would be a suitable parameter to monitor for insertion. In the present study, a low-cost pressure-indicating sensor film (PISF), Pressurex-micro® Green was chosen for investigation as a method to provide feedback on MN insertion. Consisting of a micro-encapsulated colour-forming material and a colour developing material, a red impression is formed following the application of a minimum pressure of 18.6 Ncm^−2^ between two contacting, mating or impacting surfaces. Microscopic pigmented particles adhered to the donor substrate are attracted to the chemically surface-treated receiver sheet where, upon application of pressure and separation of the two layers, micro-shear forces compel the particles to dislodge the pigment, leaving an impression. Designed with low contact pressures in mind, the colour intensity of the resulting image indicates the relative amount of pressure applied to it. To this end, the greater the pressure exerted on the film, the darker the intensity colour produced on the donor film. The two contacting, mating or impacting surfaces in this case refer to the MN array and the thumb used for application. By attaching such a film to the reverse of the MN array, upon application of pressure by the thumb, a colour change should occur, qualitatively highlighting the force employed. Previous work has shown that a force of 11.0 N is sufficient to apply a MN array of 1 cm^2^ (13), although a degree of MN penetration was demonstrated to occur from forces as low as 4.4 N/array. A minimum pressure requirement of 18.6 Ncm^−2^ for a response has been chosen to ensure an appropriate depth of MN insertion, while also providing an appropriate margin of safety, in order to guarantee MN insertion. The current study, therefore, aims to assess the combination of a PISF with hydrogel-forming MNs, as an indicator of pressure application and, hence, successful insertion, using optical coherence tomography (OCT) as a tool to confirm penetration.

## MATERIALS AND METHODS

### Chemicals

Gantrez® AN-139, a copolymer of methyl vinyl ether and maleic anhydride (PMVE/MA, molecular weight = 1,080,000) was a gift from Ashland, Kidderminster, UK. Poly(ethylene glycol) (PEG, molecular weight 10,000 Da) was purchased from Sigma-Aldrich, Steinheim, Germany. Millipore water was used throughout the study, while all other chemicals used were of analytical reagent grade. Moisture-impermeable, heat-sealable poly(ester) foil was purchased from Transparent Film Products Ltd., Newtownards, UK. Pressurex-micro® Green tactile pressure-indicating sensor film was purchased from Sensor Products Inc., Madison, New Jersey, USA. Elastoplast® Invisible Protection plasters were obtained from Beiersdorf, Hamburg, Germany.

### Fabrication of Hydrogel-Forming MN Arrays

A Gantrez® AN-139 stock solution was prepared by adding the required mass of Gantrez® AN-139 to ice-cold deionised water, followed by stirring and heating at 95°C until a clear gel was obtained, due to hydrolysis of the anhydride form of the copolymer to the corresponding acid (14). Upon cooling, the gel was readjusted to the final concentration by addition of an appropriate amount of deionised water. MN arrays were then prepared from aqueous blends containing 15% w/w Gantrez® AN-139 and 7.5% w/w PEG 10,000 as previously described (5). The blend (500 mg) was poured into laser-engineered silicone micromoulds composed of 361 (19 × 19) needles perpendicular to the base and of conical shape. The needles were each 600 μm high, with base width of 300 μm and 50 μm interspacing, arranged on an area of 0.49 cm^2^, with a MN-free border giving a final array area of 1.0 cm^2^. The filled moulds were centrifuged for 15 min at 2205 g and dried at ambient temperature for 48 h. MNs were cross-linked (esterification reaction) by heating at 80°C for 24 h and the sidewalls formed by the moulding process removed using a heated blade. Each MN array was affixed to an Elastoplast® Invisible Protection plaster for application purposes. A PISF (25 × 40 mm) was then attached using double-sided adhesive tape (3 M, Carrickmines, Ireland), to the reverse of the plaster. Control MN arrays did not have the addition of the PISF, consisting solely of the MN array and the plaster. Once assembled, each MN patch was individually packaged using moisture-impermeable heat-sealable poly(ester) foil and stored in the laboratory under ambient conditions until required.

### Pilot ***In Vitro*** Insertion Studies

Initial investigations were conducted using excised neonatal porcine skin, previously described as a suitable model for human skin (15), and a TA.XT-Plus Texture Analyser (Stable Microsystems, Haslemere, UK) for insertion of the MN and PISF combination. Full thickness neonatal porcine skin was obtained from stillborn piglets and excised <24.0 h after birth. Full thickness skin (~0.5 mm) was then stored in aluminium foil at −20.0°C until further use. Two sections of skin were placed together, with the dermal sides in contact, such that the *stratum corneum* surface was exposed on each side, resulting in a total skin thickness of approximately 1 mm, ensuring sufficient depth and support for MN insertion. MN arrays with PISF attached were gently placed on top of the neonatal porcine skin and this set-up was positioned under the probe of the Texture Analyser, the contacting surface of which had an area of 1 cm^2^. MN insertion was performed using three forces, 20 N, 30 N and 50 N, each held for 30 s. The pre-test and post-test speeds were both set at 0.5 m/s and the trigger force at 0.049 N. Inserted MN arrays were viewed *in situ* using an EXT1301 VivoSight® OCT microscope and the resultant 2D images analysed using the imaging software, ImageJ® (National Institute of Health, Maryland, USA). The swept-source Fourier domain OCT system has a laser centre wavelength of 1,305 ± 15 nm, facilitating real-time high resolution imaging of the upper skin layers (<7.5 μm lateral and < 10 μm vertical resolution). The scale of the image files obtained was 1.0 pixel = 4.2 μm, thus allowing accurate measurements of the depth of MN penetration. For each insertion, data was presented as means (± S.D.) of 10 replicate measurements of individual MN penetration depth, where the measured MNs were selected at random from the 361 penetrating MNs in each array. Following each insertion, the receiver layer of the PISF was removed and subjected to colorimetric analysis. The same set-up was followed for manual application, with insertion of the MNs into the excised skin performed by the researcher (SON) using thumb pressure, in place of the Texture Analyser.

### Volunteer Recruitment

A detailed protocol was written for the human insertion study and ethical approval from the School of Pharmacy Ethics committee was granted prior to study commencement. Twenty healthy volunteers (ten females and ten males) were recruited from the final year undergraduate pharmacy population at the School of Pharmacy, Queen’s University of Belfast, *via* an email circular. Students who had previous experience of MN research or any pre-existing skin conditions were excluded from the study. All volunteers were fully briefed on the study and provided informed consent before proceeding. For 24 h prior to the study, subjects were asked not to use any cosmetic products on their ventral forearm.

### Volunteer Application Protocol

**Fig. 1:**
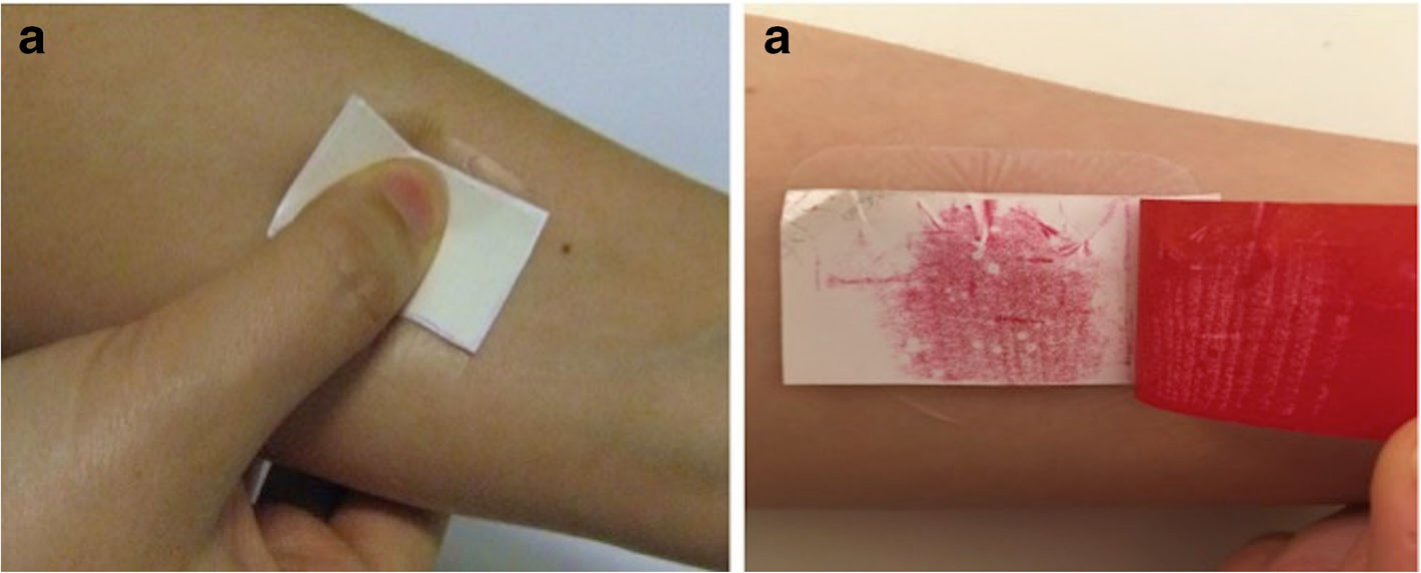
Images showing the application of thumb pressure by the subject to the MN patch on their ventral forearm (**a**) and the resulting colour change of the PISF (**b**).

Before beginning the experiment, subjects were asked to rest in the controlled environment of the study room for 15 min, in order to acclimatise to the experimental conditions of 20°C and a relative humidity of 45 ± 5%. During this time, they were asked to read the study information sheet and to complete the consent form. Each subject applied two MN arrays in total. One MN array had the PISF included in the patch and the other did not, with the order of application of the two MN arrays varied equally between the subjects. Two areas were marked on the ventral forearm of the subjects (~1.5 cm^2^) and numbered 1 and 2. These areas were then cleansed with an antiseptic wipe. The subject was presented with the first MN package, and asked to apply the array to the area labelled number 1 on their ventral forearm. Instruction on application was provided in the form of a patient information leaflet and counselling by the researcher (EMA). This strategy was informed by previous research (7) and adapted for this particular study to account for the incorporation of the PISF in the MN array. In brief, the MN package was opened and after carefully peeling off the protective backing of the plaster, the MN patch was applied to the ventral forearm. The subjects were then instructed to apply firm pressure to the reverse of the MN patch using their thumb for 30 s, ensuring the pressure was applied downwards, in the direction of the skin. If the PISF was present, the subjects were able to observe the colour change in response to the pressure applied, as shown in Fig. 1. The PISF layer, if present, was then removed for further analysis. Whilst inserted in the forearm, OCT was used to visualise the MNs *in situ* for determination of penetration depth. A VivoSight® high-resolution OCT Scanner with a lightweight and manoeuvrable handheld probe (Michelson Diagnostics Ltd., Kent, UK) was used for this purpose. The resultant 2D images from OCT scans were analysed as detailed for the insertion into excised porcine skin, with 10 replicate measurements of individual MN penetration depth recorded. The subject was then able to remove the MN array from their arm. The second MN application was performed following the same procedure, with the array being applied in this case to the location on the arm labelled number 2.

### Colorimetric Analysis

The receiver layers of the PISF from all MN applications, both *in vitro* and *in vivo*, were retained and scanned to produce digital images. The scanned image of each individual receiver layer was then analysed using the Interactive 3D Surface Plot tool from ImageJ® software. This tool establishes an arbitrary scale related to the colour intensity, with the varying degrees of colour indicating the pressure, rather than a specific value. The surface plots generated are 3D, where the X- and Y-axes correspond to the area of the film and the Z-axis indicates colour intensity, providing a visual interpretation of the pressure distribution over the 1.5 cm^2^ area of the film. Elevated peaks, on the 3D plot, indicate that a greater pressure was applied to that area.

### Questionnaire

In order to investigate the views and opinions of the volunteers regarding MNs, a short, structured questionnaire was developed. The patient satisfaction and preference questionnaire (PASAPQ), which considers performance, convenience, preference and willingness to continue, was consulted to assist in producing the questionnaire for this study (16). The questionnaire used the Likert Scale (strongly negative – strongly positive), to assess the degree of agreement with a number of statements regarding the insertion process and their overall perception of MN technology. Volunteers were also asked to state their preference (if any) for the MN array, with the PISF or without. Once designed, the questionnaire was piloted with researchers in the School of Pharmacy, who were also registered pharmacists, to ensure clarity of questions and ease of completion. All subjects were then asked to complete this questionnaire at the end of the study, following MN application.

### Statistical Analysis

Where appropriate, data was analysed using a *t-*test or a One-Way Analysis of Variance (ANOVA) with *post-hoc* comparison performed using Tukey’s HSD test. In all cases, *p* < 0.05 denoted significance.

## RESULTS

### Pilot *In Vitro* Insertion Studies

**Fig. 2:**
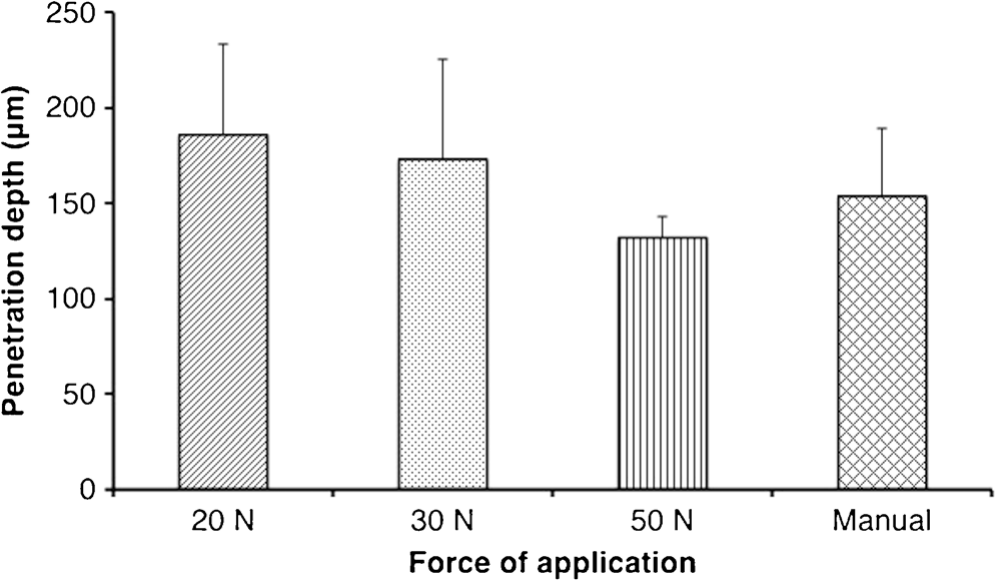
MN penetration depth in excised neonatal porcine skin applied using the Texture Analyser at varying forces and manual thumb pressure (Means ± S.D., *n* = 5).

The penetration depths of MNs, with attached PISF, inserted into excised neonatal porcine skin using three different forces as applied by the Texture Analyser are displayed in Fig. 2. For comparative purposes, the application of a MN array into the porcine skin was also completed using thumb pressure, as applied by the researcher (SON). The penetration depth decreased as the force of application was increased, contrary to what may be predicted. However, there was no significant difference overall in the penetration depths produced by 20 N, 30 N, 50 N or manual application (*p* = 0.20). The receiver layers of the PISF were retained after each application into the neonatal porcine skin at the varying forces, scanned and subjected to colorimetric analyses. Table I includes one representative example of the scanned receiver layer and interactive surface plot 3D image generated for each force employed for MN insertion into porcine skin. As would be expected from the specifications of the PISF, a general trend can be established that, the higher the force, the greater the transfer of red colour from the donor layer to the receiver layer. The areas of the film subjected to higher pressures, revealed a darker impression of colour in the receiver layer, supported by an increase in the peak heights on the surface plot graph. It follows, therefore, that as the force increases, the predominant colours of the surface plots relate to the upper end of the arbitrary scale. It can be seen that the distribution of the red colour is not uniform when the Texture Analyser probe is used for application, with darker red areas, due to the sides of the MN array, distinguishable in all cases. When the force was applied manually, however, a difference in the pattern of colour formed on the receiver layer was noticeable, with a more uniform colour distribution observed.

**Table I:**
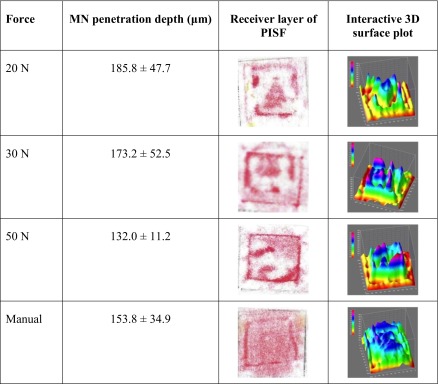
Mean Penetration Depths (*n* = 5) and Representative Images of the Receiver Layer of the PISF and the Interactive 3D Surface Plot Generated, Following MN Insertion into Excised Neonatal Porcine Skin Using the Texture Analyser at 20 N, 30 N, 50 N and Manual Thumb Application

### Insertion by Human Subjects

Twenty healthy volunteers (10 male and 10 female) aged between 21 and 23 years old completed the study. MN application was well-tolerated by all subjects and no adverse reactions were noted. All subjects were able to successfully self-apply MN arrays, with penetration of the *stratum corneum* confirmed in each case by OCT. Penetration depth was measured for the insertion of each MN array, both with and without the PISF incorporated (Table II). There was no significant difference in the penetration depth (*p* = 0.17) obtained with each MN array, as self-applied by the subjects, regardless of whether or not the PISF was included. Colorimetric analyses revealed uniform transfer of the red colour from the donor to the receiver layer, resulting in relatively even 3D surface plots, with greater consistency of colour over the full area of the film than observed in the *in vitro* tests. Table III includes the penetration depth, alongside the scanned receiver layer and 3D surface plot generated for three subjects, upon MN insertion, as examples of the images obtained from each participant.

**Table II:** MN Penetration Depth when Self-Applied in Human Subjects, with and Without the Incorporation of a PISF (Means ± S.D., *n* = 10)

	MN penetration depth (μm)
With PISF	245.2 ± 65.8
Without PISF	228.1 ± 53.7

**Table III:**
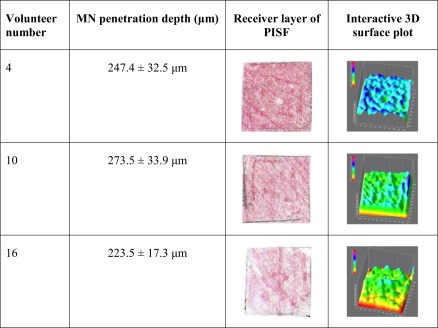
Mean Penetration Depths and Representative Images of the Receiver Layer of the PISF and the Interactive 3D Surface Plot Generated for Three Volunteers, Following Self-Application of MN Arrays into the Ventral Forearm

### Questionnaire

**Fig. 3:**
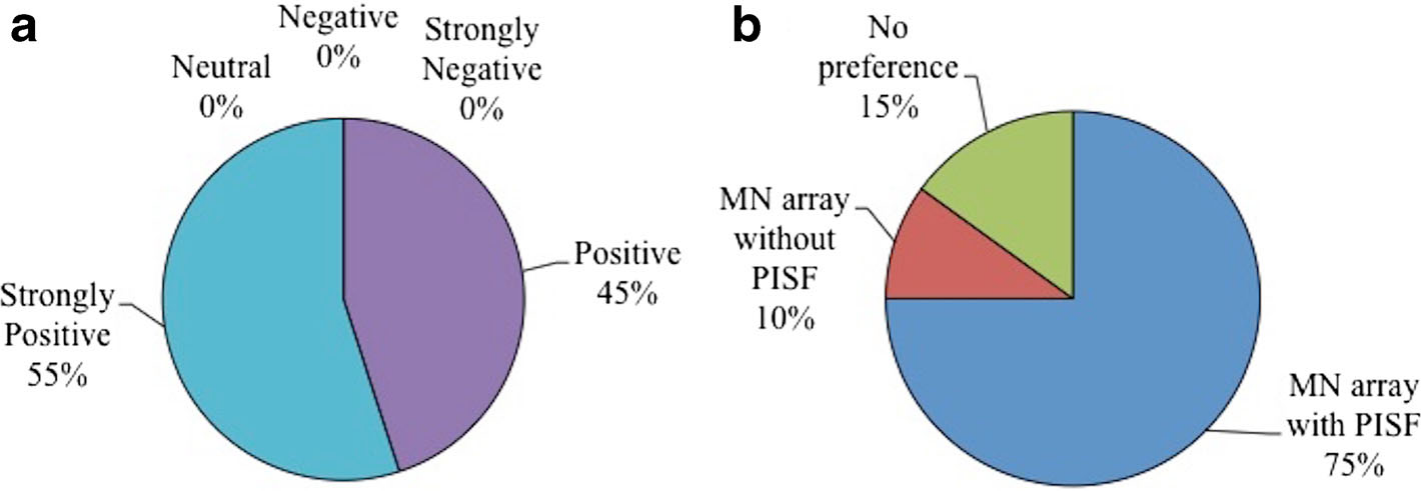
Subjects’ opinions regarding MN technology in general (**a**) and preference for MN arrays with or without PISF (**b**), following MN application.

The responses to the questionnaire that was completed by all participants post-application are summarised in Table IV. As summative questions, subjects were asked their opinion on MN technology in general and whether they had a preference for the MN array applied in combination with a PISF or without. All were positive or strongly positive about MN technology and, in total, 75% expressed a preference for the MN array with the PISF incorporated, as displayed in Fig. 3.

**Table IV:** Human Subjects’ Rating of MNs and the Application Procedure, According to a Likert Scale (*n* = 10)

Scenario	Very satisfied	Satisfied	Neither satisfied or dissatisfied	Dissatisfied	Very dissatisfied
Application procedure	14	6	0	0	0
Applied dose would enter the body	9	8	3	0	0
Works reliably	9	8	3	0	0
Ease of application	14	5	0	1	0
Counselling & patient information leaflet	18	1	1	0	0

## DISCUSSION

There are a number of factors considered central to the potential success of MNs as a commercially available pharmaceutical product, including their ability to perform as designed, their ability to be used correctly (with reproducible insertion) and, also, their acceptance by both patients and healthcare professionals (17). Initial exploration of the views and opinions regarding MNs, for both drug delivery and monitoring purposes, have yielded positive results, with the majority recognising significant advantages to the use of the platform (8–11). Encouragingly, people have expressed their willingness to self-apply MNs, without the intervention of a healthcare professional, indeed, highlighting this option as one of the potential benefits. Concerns have, however, been noted regarding the uncertainty which may be associated with MN use, in terms of understanding that it has been correctly applied, particularly associated with the force required for MN insertion. One option to address this is the use of an applicator device for insertion, providing a calibrated force for each application. However, such an approach does not necessarily provide reassurance to the user and is likely to result in a more complex product, which may inhibit patient adherence (17). Another, more promising, proposed solution is the incorporation of a method of feedback into a MN patch, designed to respond and inform the user of successful MN application, thus ensuring drug delivery or equally, sample collection, if being used for monitoring purposes. The use of a method of feedback to ensure reproducible insertion is preferable to an applicator, in order to mimic the conventional transdermal patches already on the market and capitalise on preconceived positive beliefs relating to such existing dosage forms. As proven by a previous study conducted by Donnelly *et al.* (7), feedback such as this is not necessary for appropriate insertion of MNs, reporting reproducible self-application of hydrogel-forming MNs, assisted by a patient information leaflet and pharmacist counselling. However, input from the end-user has been previously observed to be closely related to the acceptance of a pharmaceutical product once commercially available, with evidence linking the oversight of potential patient perspectives to the failure or poor uptake of many health innovations, including drug delivery devices (18). It is, therefore, important to acknowledge and act upon potential issues such as this, to assist in a smooth transition to clinic, enabling the end-user to derive the greatest benefit from the final MN product. Indeed, it is also thought it may be necessary to include some form of ‘dosing indicator,’ similar to that described, to gain acceptance from regulatory authorities (e.g., Food and Drug Administration, European Medicines Agency) (17). This study, therefore, investigated the ability of a PISF to act as a feedback mechanism, when combined with a MN array, specifically considering the colour change that occurred and the resulting needle penetration depth.

The use of the Texture Analyser for insertion of MNs combined with a PISF into excised neonatal porcine skin was designed as an *in vitro* precursor to *in vivo* application in human subjects. Three forces were chosen to demonstrate the potential difference in colour change occurring upon the application of increasing force and any related impact on penetration depth. The PISF used stipulates a minimum pressure of 18.6 Ncm^−2^ to cause colour transfer, hence 20 N was the lowest force chosen for investigation, applied using a 1 cm^2^ probe. A change in colour from white to red was noticeable on all receiver layers following application of force by the texture analyser. In each case, needle insertion into the porcine skin was also achieved, with mean penetration depths as detailed in Table I. This demonstrates the ability of the PISF to successfully indicate the application of force to the MN array and, hence, the insertion of needles into the skin. There was, however, incomplete contact between the Texture Analyser and the MN array, due to the rigidity of the metal probe and the potentially uneven surface of the skin, which was reflected in the scattered colour pattern produced. In contrast, the use of thumb pressure for MN insertion into excised neonatal porcine skin created a more uniform distribution of colour, at a force estimated to be equivalent to approximately 20 N, based on findings reported by Larreñata *et al.* (12). This difference in response is likely due to the ability of the thumb to conform around the MN array and the skin below, thereby distributing the pressure over the full area of the array. Such a factor is likely to be of greater importance *in vivo*, considering the contours of the body. Despite the variability observed in the colorimetric analyses of the different *in vitro* applications, there was no significant difference in the penetration depths of each MN array applied at different forces (*p* = 0.20). It can, therefore, not be established from the *in vitro* test in this case, if the application of a greater force and, hence, higher pressure will result in deeper MN penetration. This is contrary to previous reports that have found a significant enhancement in the depth of penetration into skin upon increase of the force used for application (13). The MNs used in the present study, however, were of a different geometry to that described (19 × 19 in comparison to 11 × 11) and, therefore, display different insertion properties. Further to this, the forces employed for application were significantly higher in the current study. At greater forces, less differentiation between penetration depths may be expected, due to a ‘bed of nails’ effect, limiting needle penetration (19), a phenomenon particularly of note with the high needle density employed in this study.

Human subjects applied two MN arrays to themselves as part of this study, one array with a PISF incorporated and one without, to act as a comparator. The aim herein was simply to verify if application *in vivo* using thumb pressure was sufficient to provoke a colour change of the PISF, thereby providing a suitable level of feedback for the user, whilst also confirming MN insertion. All subjects were able to successfully self-apply the MN arrays, following appropriate instruction, in line with previous studies investigating the ability of human subjects to use MNs (6,7). Using OCT, a mean penetration depth of 237 μm was recorded, similar to previously reported values for this particular type of MN array (20). There was no significant difference between the penetration depths recorded from each of the two types of MN array i.e. with and without the PISF, demonstrating that the addition of a PISF did not affect the performance of the subject or indeed, the insertion of the needles. In all cases, when the PISF was incorporated, there was a colour change following application of thumb pressure to the array, verifying that the force applied by the human subjects was enough to cause the transfer of colour from the donor to the receiver layer of the film. Considered in context, with the OCT data discussed previously, it can then also be stated that sufficient force was applied to cause needle penetration into the skin. Although the exact force applied by each subject was unknown, due to the response, it can be assumed that the resultant pressure was above 18.6 Ncm^−2^, the threshold for a colour change. As a visual aid for the user, the feedback mechanism, therefore, fulfilled its purpose of confirming the application of sufficient force for MN insertion. The participants reinforced the success of the PISF, with 75% expressing a preference for the combination of the MN array with the PISF incorporated, following application of MNs with and without it. This is in agreement with previous studies, which have highlighted the desire of both potential end-users and prescribers to receive some form of feedback upon MN application, as an assurance of correct use (8–11). This outcome provides important information for the translation of MN technology from the laboratory to a viable commercial product. The addition of a PISF is likely to assist in the roll-out of a MN product to a mainstream market, by enhancing patient acceptability and maximising ease of use. Indeed, in certain clinical situations, the use of a PISF would be particularly useful to confirm MN insertion. For example, the delivery of high doses of low potency drugs using polymeric MNs has recently been realised, proposing a patch size of up to 30 cm^2^ to achieve therapeutic levels of a low molecular weight drug in human (4,21,22). It is envisaged that such a larger patch size would be self-applied using two or three fingers, rather than the thumb, as described for 1 cm^2^ arrays. In these cases, a method of feedback, such as a colour change, upon MN insertion would likely be a vital component of the patch to confirm pressure application across the complete surface of the MN array. A PISF is, therefore, likely to have great impact in the future translation of MN research to commercial products, in clinical use. In combination with standard measures, such as a patient information leaflet and pharmacist counselling, a method of feedback may assist people in applying MN arrays safely and effectively, providing further evidence for the feasibility of MN self-application.

## CONCLUSION

For the first time, this study shows how the incorporation of a simple, low-cost PISF can ensure MN insertion in human subjects, providing a level of assurance regarding appropriate use. A colour change occurred in all cases, alongside MN penetration, demonstrating that a PISF can provide visual feedback to the user upon MN application. Further to this, three quarters of the subjects involved declared a preference for the MN and PISF combination, following application of both it and a control array. This study, therefore, provides convincing evidence as to the use of a feedback mechanism, specifically a PISF, for confirmation of successful MN self-application.

## ABBREVIATIONS

EMA: Emma McAlister
MN: Microneedle
PISF: Pressure-indicating sensor film
SON: Shannon O’Neill

## References

[1] Henry S, McAllister DV, Allen MG, Prausnitz MR (1998). Microfabricated microneedles: a novel approach to transdermal drug delivery. J Pharm Sci.

[2] Larraneta E, Lutton REM, Woolfson AD, Donnelly RF (2016). Microneedle arrays as transdermal and intradermal drug delivery systems: materials science, manufacture and commercial development. Mater Sci Eng R.

[3] McCrudden MTC, Alkilani AZ, Courtenay AJ, McCrudden CM, McCloskey B, Walker C (2014). Considerations in the sterile manufacture of polymeric microneedle arrays. Drug Deliv Transl Res.

[4] Donnelly RF, McCrudden MTC, Zaid Alkilani A, Larrañeta E, McAlister E, Courtenay AJ (2014). Hydrogel-forming microneedles prepared from “super swelling” polymers combined with lyophilised wafers for transdermal drug delivery. PLoS One.

[5] Donnelly RF, Thakur RRS, Garland MJ, Migalska K, Majithiya R, McCrudden CM (2012). Hydrogel-forming microneedle arrays for enhanced transdermal drug delivery. Adv Funct Mater.

[6] Norman JJ, Arya JM, McClain MA, Frew PM, Meltzer MI, Prausnitz MR (2014). Microneedle patches: usability and acceptability for self-vaccination against influenza. Vaccine.

[7] Donnelly RF, Moffatt K, Alkilani AZ, Vicente-Pérez EM, Barry J, McCrudden MTC (2014). Hydrogel-forming microneedle arrays can be effectively inserted in skin by self-application: a pilot study centred on pharmacist intervention and a patient information leaflet. Pharm Res.

[8] Birchall JC, Clemo R, Anstey A, John DN (2011). Microneedles in clinical practice—an exploratory study into the opinions of healthcare professionals and the public. Pharm Res.

[9] Mooney K, McElnay JC, Donnelly RF (2014). Children’s views on microneedle use as an alternative to blood sampling for patient monitoring. Int J Pharm Pract.

[10] Mooney K, McElnay JC, Donnelly RF (2015). Paediatricians’ opinions of microneedle-mediated monitoring: a key stage in the translation of microneedle technology from laboratory into clinical practice. Drug Deliv Transl Res.

[11] Caffarel-Salvador E, Tuan-Mahmood T-M, McElnay JC, McCarthy HO, Mooney K, Woolfson AD (2015). Potential of hydrogel-forming and dissolving microneedles for use in paediatric populations. Int J Pharm.

[12] Larrañeta E, Moore J, Vicente-Pérez EM, González-Vázquez P, Lutton REM, Woolfson AD (2014). A proposed model membrane and test method for microneedle insertion studies. Int J Pharm.

[13] Donnelly RF, Garland MJ, Morrow DIJ, Migalska K, Thakur RRS, Majithiya R (2010). Optical coherence tomography is a valuable tool in the study of the effects of microneedle geometry on skin penetration characteristics and in-skin dissolution. J Control Release.

[14] McCarron PA, Woolfson AD, Donnelly RF, Andrews GP, Zawislak A, Price JH (2004). Influence of plasticizer type and storage conditions on properties of poly(methyl vinyl ether-co-maleic anhydride) bioadhesive films. J Appl Polym Sci.

[15] Cilurzo F, Minghetti P, Sinico C (2007). Newborn pig skin as model membrane in *in vitro* drug permeation studies: a technical note. AAPS PharmSciTech.

[16] Kozma CM, Slaton TL, Monz BU, Hodder R, Reese PR (2005). Development and validation of a patient satisfaction and preference questionnaire for inhalation devices. Treat Respir Med.

[17] Donnelly RF, Woolfson AD (2014). Patient safety and beyond: what should we expect from microneedle arrays in the transdermal delivery arena?. Ther Deliv.

[18] Heinemann L (2008). The failure of Exubera: are we beating a dead horse?. J Diabetes Sci Technol.

[19] Olatunji O, Das DB, Garland MJ, Belaid L, Donnelly RF (2013). Influence of array interspacing on the force required for successful microneedle skin penetration: theoretical and practical approaches. J Pharm Sci.

[20] Donnelly RF, Mooney K, McCrudden MTC, Vicente-Pérez EM, Belaid L, González-Vázquez P (2014). Hydrogel-forming microneedles increase in volume during swelling in skin, but skin barrier function recovery is unaffected. J Pharm Sci.

[21] McCrudden MTC, Alkilani AZ, McCrudden CM, McAlister E, McCarthy HO, Woolfson AD (2014). Design and physicochemical characterisation of novel dissolving polymeric microneedle arrays for transdermal delivery of high dose, low molecular weight drugs. J Control Release.

[22] Kearney M-C, Caffarel-Salvador E, Fallows SJ, McCarthy HO, Donnelly RF (2016). Microneedle-mediated delivery of donepezil: potential for improved treatment options in Alzheimer’s disease. Eur J Pharm Biopharm.

